# Scientific Research Trend on Guided Tissue Regeneration: A Bibliometric Analysis

**DOI:** 10.1055/s-0044-1791529

**Published:** 2024-11-21

**Authors:** Abdulkareem Abdullah Alhumaidan, Beenish Fatima Alam, Asim Alsuwaiyan, Eman Ahmed Aljoghaiman, Mohammad Helmi, Saqib Ali

**Affiliations:** 1 Department of Preventive Dental Sciences, College of Dentistry, Imam Abdulrahman Bin Faisal University, Dammam, Saudi Arabia; 2 Department of Oral Biology, Bahria University Dental College, Karachi, Pakistan; 3 Periodontics Unit, Dental Department, King Fahd Military Medical Complex, Dhahran, Saudi Arabia; 4 Department of Periodontics and Community Dentistry, College of Dentistry, King Saud University, Riyadh, Saudi Arabia; 5 Department of Biomedical Dental Sciences, College of Dentistry, Imam Abdulrahman Bin Faisal University, Dammam, Saudi Arabia

**Keywords:** periodontal tissue regeneration, Scopus, VOSviewer, periodontitis, membrane

## Abstract

**Objectives** Guided tissue regeneration (GTR) is a widely used technique in contemporary dentistry which helps achieve regeneration of periodontal tissues. This study aims to identify leading countries, authors, institutes, journals, scientific publications, and mostly used keyword regarding role of GTR in treatment for periodontal disease using the Scopus database.

**Materials and Methods** A well-curated search through Scopus database for significant literature related to GTR published between 1987 and 2023 was performed. Bibliographical data which comprised of abstracts, title, keywords, references, citations, and other relevant information were composed. The data was analyzed using MS Excel and VOSviewer.

**Results** Scientific literature on GTR was manually scrutinized, and 308 paper were analyzed using the Scopus database. The first paper on GTR was published in 1987. *Journal of Periodontology* was identified as the leading journal, while the United States and Italy were the top contributing countries. University of Sienna was the most productive organization. Roberto Pontoriero was identified as the highly cited author. A highly cited scientific paper was published by Pintippa Bunyaratavej in 2001.

**Conclusion** The present bibliometric study gives useful information related to the total number of scientific articles published from 1987 to 2023. A rising trend of scientific publication was identified which continued followed by a notable decline after 2004. The analysis also recognized the United States and University of Sienna, from Italy as most active country and organizations, while the *Journal of Periodontology* as the leading journal.

**Clinical Relevance** This study may assist in continuing education and evidence-based practice for clinicians and new researchers by providing knowledge and aiding literature searches in the domain of GTR used in treatment for periodontal conditions.

## Introduction

Periodontitis, defined as chronic multifactorial inflammatory disease, has been linked to dysbiotic plaque biofilms. 1 2 Periodontal degeneration causes accumulation of dead tissues and leads to inflammatory reactions in the gingiva and in some cases causes periodontitis. 3 Conservative treatment comprises of scaling and root planning and using antibiotics. In addition, root conditioning, conservative therapy, bone substitutes and grafts, guided tissue regeneration (GTR), and combination of bone grafts and GTR help in periodontal tissue regeneration. 4 5 6

Hurley et al used GTR for the first time back in the 1950s. 7 GTR for periodontal tissue regeneration has been used since the 1980s. 7 8 GTR repairs the periodontal attachment by limiting epithelial cell proliferation and allows periodontal ligament (PDL) and bone to proliferate. 2 9 It used materials for bone grafting and membrane barriers which regenerated the new attachment. 10 Furthermore, placing a physical barrier between the tissues and degraded area prevents the flap from altering its position and prevents tissues from encountering this space. 11 12 To act as a barrier membrane, GTR membrane must be biocompatible, nontoxic, safe, mechanically stable, tissue integrating, and clinically controllable. 13 14

Scientific metrics have been used to identify the influence and outcome of research. Bibliometric analysis explores data, gathers varying trends over period, identifies areas of interest, leading authors, institutes, countries, journals, and provides an overview of research. 15 16 This trend of bibliometric analysis in scientific publications has increased in recent years, notably in the field of periodontology, dental polymers, salivary biomarkers, coronavirus disease 2019, dental implants, and mineral trioxide aggregate. 16 17 18 19 20 21

Numerous published bibliometric analyses have focused on various domains which comprises of photodynamic therapy, periodontal regeneration, bone regeneration, and regeneration related to periodontal surgery. 22 23 24 25 26 However, none of the previously conducted bibliometric analysis had assessed the role of GTR as a treatment option for periodontal disease. Hence, the current analysis aims to identify leading countries, authors, institutes, journals, scientific publications, and the most used keyword regarding role of GTR in treatment for periodontal disease.

## Materials and Methods

### Search Database

The search for relevant papers was performed on August 5, 2024, using the Elsevier's Scopus database. Because of its user-friendly features that make executing bibliometric analyses easier, Scopus is widely used by researchers seeking to perform high-quality analyses. 27 Elsevier's Scopus is frequently viewed as the most extensive scientific database, even outperforming PubMed and the Web of Science (WoS). In contrast, the WoS covers 54% of Scopus titles, while Scopus has 84% of the titles indexed in the WoS. 28 Additionally, Scopus is more dependable than Google Scholar due to its higher citation management and well-chosen indexing. 29

### Selection of Papers Using Keywords

**Fig. 1:**
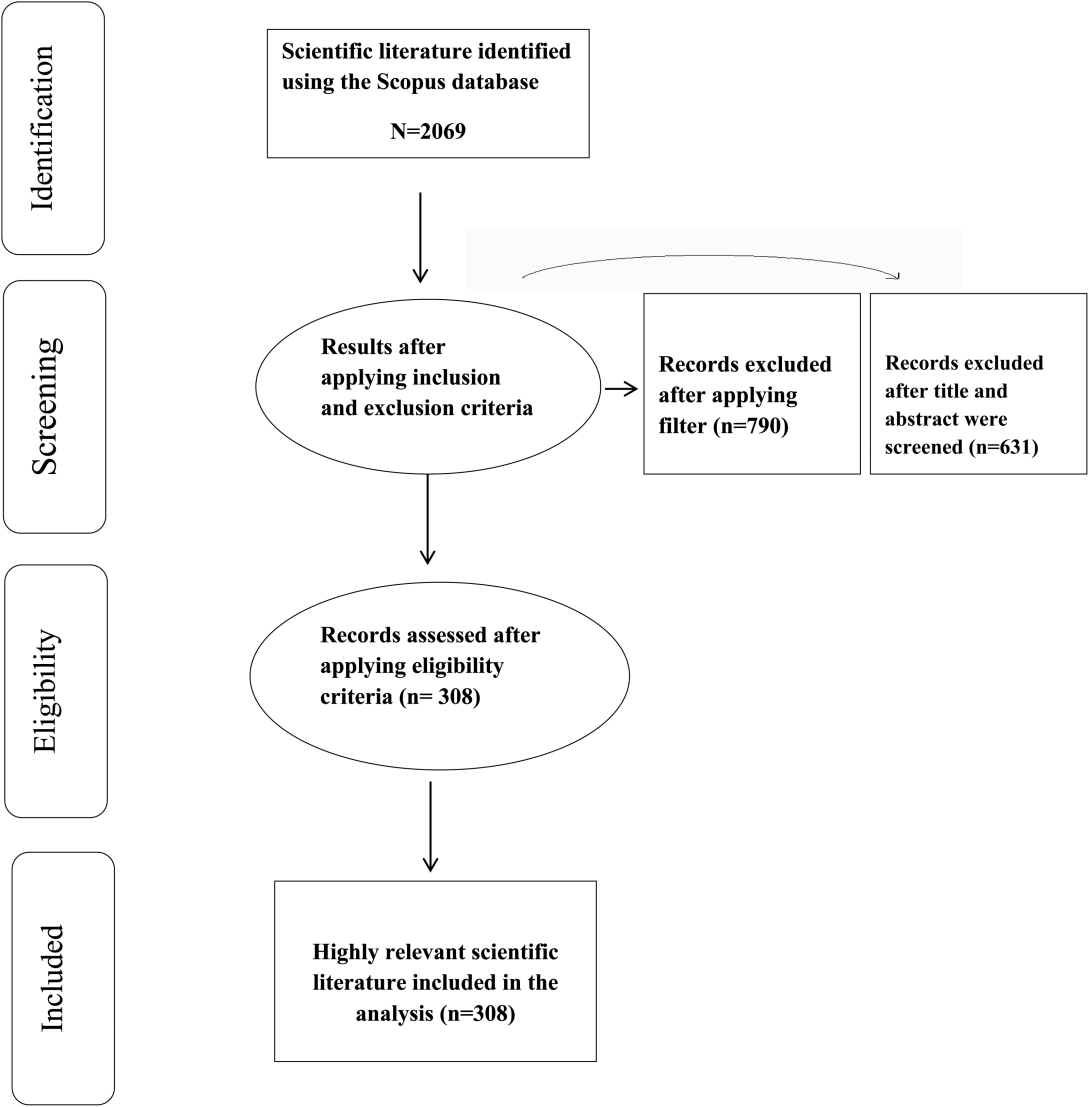
A flow diagram describing the literature search using Scopus database.

Search on Scopus was conducted and search terms “Guided tissue regeneration AND Periodontal Disease” were used to retrieve appropriate results from the Scopus database. Our search comprised of searching for titles, abstracts, and keywords. More than 2,000 articles were found during the initial search. The inclusion criteria for the study were applied, which included only English-language scientific publications, “Original article” and “Reviews” were accepted as the publication types, and “Dentistry” was chosen as the subject area. This reduced the number of articles to approximately 1,279 papers. In line with this, exclusion criteria included scientific literature not focusing on GTR, other paper types, which included case studies, case reports, and chapters from thesis and books, and publications from other fields like medicine, biochemistry, and material sciences, along with these studies focusing on animals had also been eliminated. Hence, our study included publications from the duration of 1987 to 2023 ( Fig. 1 ).

### Data Analysis

After applying the inclusion criteria, and excluding studies based on animals and case reports, the remaining approximately 648 papers were manually scrutinized and after careful evaluation of abstracts and titles, pertinent publications related to our topic were selected.

All the selected articles ( *n* = 308) were downloaded from the Scopus database in the form of comma-separated values format. The file was stored on a hard drive and further studied with the help of the VOSviewer software (v1.6.18; Center for Science and Technology Studies, Leiden University), a bibliometric software program, and later transferred as tab-delimited files, on Microsoft Excel. 30 31

The VOSviewer software can create collaborative linkages for various keywords and variables. This software helped identify leading journals, authors, countries, and frequently used keywords along with highly cited papers. VOSviewer helped generate visual maps highlighting leading institutes, journals, countries, and most used keywords. With each map the size of a bubble indicated the term's frequency of occurrence. If two terms appeared together more frequently in the publications under analysis, then two bubbles are positioned closer to one another. The average number of citations in each publication was represented by the color. Lastly, keywords which had the maximum number of occurrences were taken into consideration, and following which maps were produced. 17 21

## Result

**Fig. 2:**
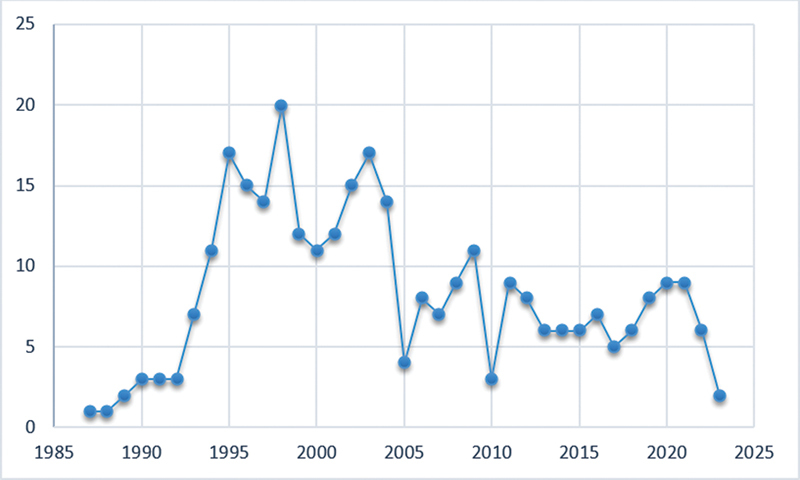
Scientific publications on guided tissue regeneration (GTR) dated from 1987 to 2023.

Fig. 2 summarizes the annual publication of scientific literature on GTR spanning from 1987 to 2023. In 1987, the first paper on GTR was published. There was relatively limited research production at first, with only one publication that appeared during 1987, and a similar pattern continued till 1992. Though with time, the number of scientific publications and citations increased gradually. From 1993 onwards, the number of publications increased to 7, while in the year 1995, number of publications increase to 17 in number. In 1998, a significant boost in the number of research articles ( *n* = 20) was observed. This trend of scientific publications continued till the year 2004. Following which, there was a decline in the number of research publications in the year 2005 when only 4 papers were published. A slight increase in scientific literature was observed from 2019 with a decline in the number of scientific publications noted in the upcoming years which continued till year 2023 ( Fig. 2 ).

**Fig. 3:**
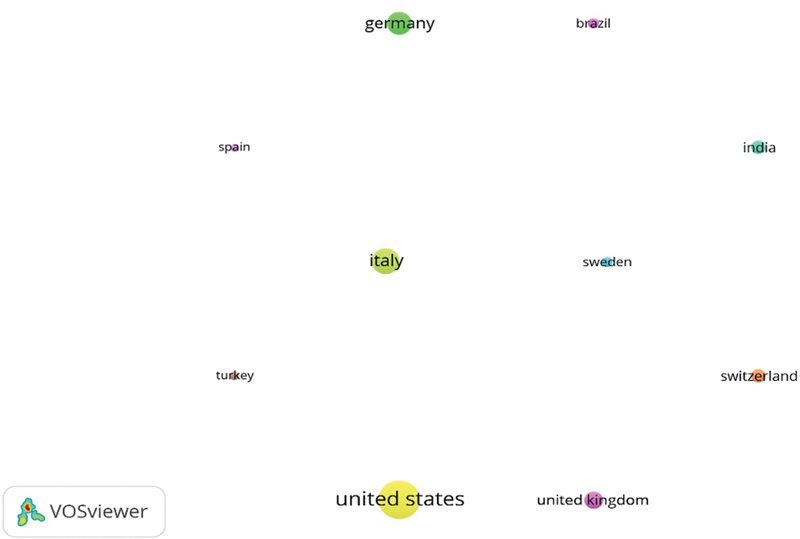
Most productive countries.

Table 1 ascertained the most active countries which had produced the highest number of scientific literatures related to GTR. From a total of 59 countries, leading top 10 countries had been identified which published at least 10 papers related to GTR. The United States was identified as the leading country which had published the highest number of articles, *n* = 91, followed by Italy ( *n* = 53), Germany ( *n* = 43), and Switzerland ( *n* = 21) ( Fig. 3 ).

**Table 1:** Leading countries

Country	Publications	Citations	Citation index
United States	91	5,012	55.07
Italy	53	4,123	77.79
Germany	43	1,930	44.88
Switzerland	21	1,495	71.19
United Kingdom	29	1,905	65.68
Sweden	15	996	66.40
Spain	10	814	81.40
Brazil India Turkey	15 21 11	534 282 266	35.60 13.42 24.18

With respect to the countries which have attained the highest number of citations for their contribution by scientific publication, the United States attained the highest number of citations (5,012), followed by Italy (4,123), Germany (1,930), and the United Kingdom (1,905). Regarding the citation index, publications from Spanish authors (81.40) had the highest citation index, despite publishing only 10 papers, followed by Italy (77.79) and Switzerland (71.19) ( Table 1 ).

**Fig. 4:**
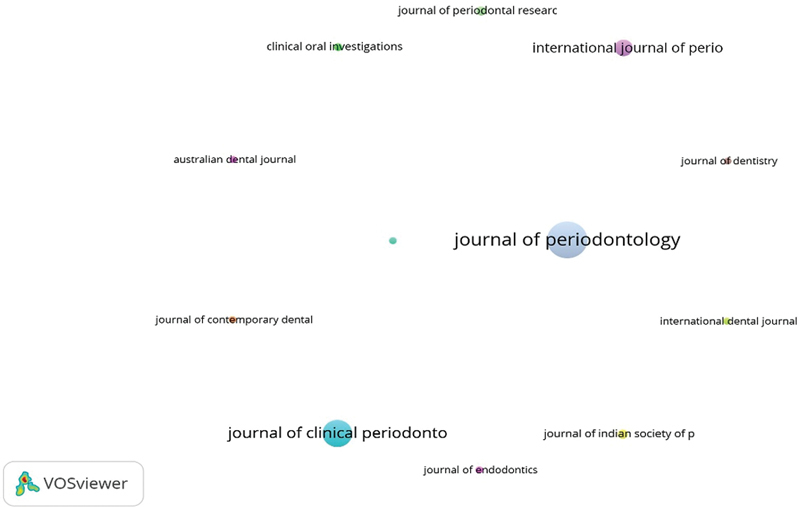
Leading journals.

Prolific journals which had published minimum of 4 papers on GTR are listed in Table 2 . *Journal of Periodontology* published the highest number of papers ( *n* = 115), while the *Journal of Clinical Periodontology* , *International Journal of Periodontics and Restorative Dentistry* , and *Journal of Indian Society of Periodontology* published *n* = 62, 24, and 8 papers, respectively ( Fig. 4 ).

**Table 2:** Highly ranked journals

Journal	Total publication	Total citation	Impact factor	Quartile	Publisher
*Journal of Periodontology*	115	7,487	4.2	1	Wiley
*Journal of Clinical Periodontology*	62	4,427	5.8	1	Wiley
*International Journal of Periodontics and Restorative Dentistry*	24	592	1.3	2	Quintessence
*Journal of Dentistry*	4	399	4.8	1	Elsevier
*Journal of Periodontal Research*	7	196	3.4	1	Blackwell Munksgaard
*Australian Dental Journal*	4	147	1.9	2	Wiley
*Dental Clinics of North America*	4	118	–	0	W.B. Saunders Ltd
*Clinical Oral Investigations*	5	44	3.1	1	Springer
*Journal of Indian Society of Periodontology*	8	158	–	0	Lippincott
*Journal of Endodontics*	4	85	3.5	1	Elsevier
*International Dental Journal*	4	55	3.2	1	Elsevier
*Journal of Contemporary Dental Practice*	4	31	–	0	Jaypee Brothers Medical Publishers

Regarding the number of citations, scientific publications from the *Journal of Periodontology* were highly cited (7,487), followed by the *Journal of Clinical Periodontology* (4,427), *International Journal of Periodontics and Restorative Dentistry* (592), and *Journal of Dentistry* (399) ( Table 2 ).

Regarding the impact factor (IF), the *Journal of Clinical Periodontology* had the highest IF (5.8) which published 62 papers, followed by the *Journal of Periodontology* , IF = 4.2, *Journal of Dentistry* , IF = 4.8, and *Journal of Periodontal Research* , IF = 3.4. Moreover, 7 out of 12 journals were Q1, 2 were Q2, while 3 did not belong to any category.

**Fig. 5:**
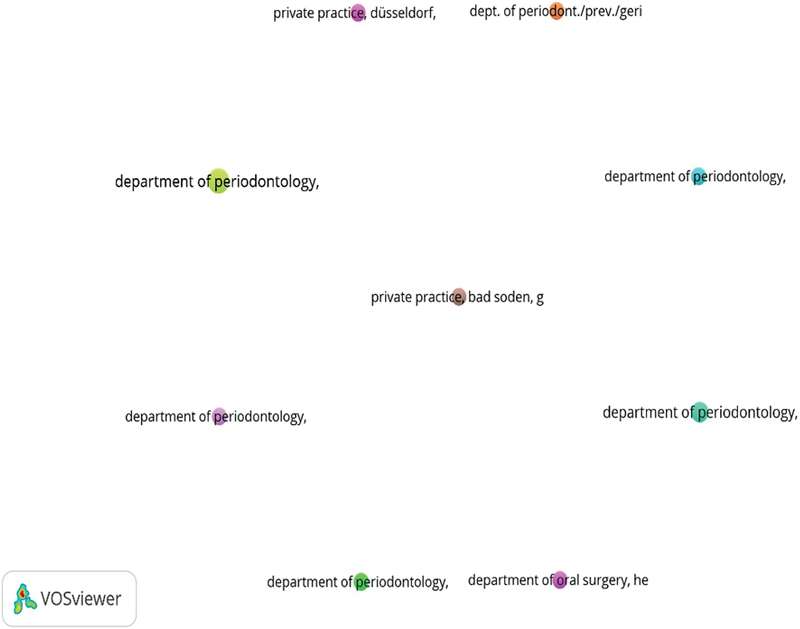
Most productive institutes.

Table 3 identified the leading institutes which produced three or more publications with regards to GTR. University of Bern was the leading institution ( *n* = 6), followed by Bologna University ( *n* = 4), while the rest of the organizations had published *n* = 3 papers each ( Fig. 5 ).

**Table 3:** Most active organizations based on citations attained

Organization	Country	Publications	Citations
University of Siena	Italy	3	510
University of Michigan	United States	3	508
University of Pennsylvania	United States	3	442
Bologna University	Italy	4	360
University College London	United Kingdom	3	310
Heinrich Heine University	Germany	3	242
University of Bern	Switzerland	6	218

Regarding the number of citations, publications from the University of Siena received 510 citations, followed by the University of Michigan, University of Pennsylvania, and Bologna University which received 508, 442, and 360 citations, respectively ( Table 3 ).

Table 4 referred to top 10 highly cited papers on GTR having more than 200 citations. These articles had attainted citations ranging from 226 to 416 and were published from 1988 to 2006. Paper by author Pintippa Bunyaratavej was cited more than 400 times. In addition, five articles were cited more than 200 times, while three were cited more than 300 times. Most of the papers were published in the *Journal of Periodontology* , followed by the *Journal of Clinical Periodontology* ( Table 4 ).

**Table 4:** Highly cited publications

Papers	First author	Main findings	Citations	Journal
Collagen membranes: a review 34	Pintippa Bunyaratavej	This review evaluated all vitro and in vitro studies which assessed the role of collagen in periodontal defects and to determine its use in various regenerative procedures	416	*Journal of Periodontology*
Periodontal plastic surgery for treatment of localized gingival recessions: a systematic review 41	Mario Roccuzzo	This systematic review evaluated the efficiency of various surgical procedures which included GTR, free gingival graft, coronally advanced flap, and connective tissue graft for treating root coverage in cases with localized gingival recessions. The study concluded that gingival recession reduced in other procedure as compared with GTR. Attachment was same in all procedures	342	*Journal of Periodontology*
The effect of membrane exposure on the outcome of regenerative procedures in humans: a meta-analysis 42	Eli E. Machtei	A systematic review was conducted to assess studies which studied the impact of early membrane exposure influencing the regenerative outcome of class II furcation and intrabony defects associated with GTR and guided bone regeneration around implant. Study observed that membrane exposure had insignificant influence on GTR	305	*Journal of Periodontology*
Effect of cigarette smoking on periodontal healing following GTR in infrabony defects 45	Maurizio S. Tonetti	This paper assessed the impact of smoking on the healing potential of tissues in deep pockets after GTR. It was observed that smokers were more inclined than nonsmokers to exhibit a reduced gain in probing attachment level due to decreased healing response following GTR	298	*Journal of Clinical Periodontology*
Periodontal regeneration of human intrabony defects. IV. Determinants of healing response 46	Maurizio S. Tonetti	The study aimed to determine factors influencing the healing potential of intrabony defects following GTR. It was noted that depth of infrabony component, along with radiological defect angle influenced that gain of tissue	275	*Journal of Periodontology*
Guided tissue regeneration versus mucogingival surgery in the treatment of human buccal gingival recession 47	Giampaolo Pini Prato	In this comparative study, surgical procedure used for treating localized recession buccally 3–8 mm was compared. Surgical procedure findings for the test and control groups were compared. It was concluded that GTR is useful procedure for gingival recession	247	*Journal of Periodontology*
Guided tissue regeneration in degree II furcation-involved mandibular molars 48	Roberto Pontoriero	The objective of this clinical trial was to assess the periodontal tissues' potential for regeneration in case of class II furcation lesions in lower molars based on guided tissue regeneration principles. It was observed that GTR proved beneficial in reducing the furcation problem	238	*Journal of Clinical Periodontology*
Flap thickness as a predictor of root coverage: a systematic review 49	Debby Hwang	This systematic review examined studies which had determined if there was a relationship between gingival thickness and root coverage outcomes. Various treatment modalities including GTR was assessed. It was seen that favorable correlation existed between root coverage and flap thickness	226	*Journal of Periodontology*

Abbreviation: GTR, guided tissue regeneration.

Table 5 enlists the first authors who had attained highest citations. Roberto Pontoriero and Pierpaolo Cortellini published *n* = 4 papers each. With respect to the number of citations, Pontoriero received highest citations for their scientific contribution (521) followed by Pierpaolo Cortellini and Randall J. Harris, who had attained 414 and 215 citations, respectively ( Table 5 ).

**Table 5:** Highly cited authors

Authors	No. of publications	Citations
Roberto Pontoriero	4	521
Pierpaolo Cortellini	4	414
Randall J. Harris	3	215
Kevin G. Murphy	2	102
Hans-Peter Müller	2	91
Peter Eickholz	3	81

**Fig. 6:**
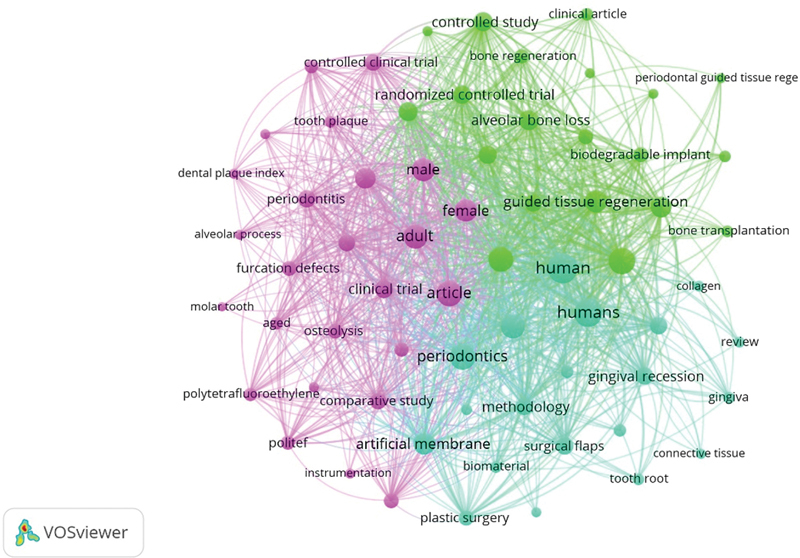
Repeatedly used keywords.

Fig. 6 presented the keyword analysis based on the scientific literature published related to GTR. Thirty times was the minimum number of occurrences for each keyword; henceforth, from a total 1,419 keywords, 65 were considered. Map generated comprised of four clusters. Each color signifies a distinct cluster, and these clusters are organized based on their link strength and occurrence. The dimension of each bubble pointed toward the type of link that existed with occurrence and link strength.

The five highly cited keywords were GTR, periodontal (occurrence = 230, total link strength [TLS] = 4,968), periodontal disease (occurrence = 201, TLS = 4,225), membrane, artificial (occurrence = 179, TLS = 4,188), PDL attachment loss (occurrence = 121, TLS = 3,075), and periodontal pocket (occurrence = 93, TLS = 2,491) ( Fig. 6 ).

## Discussion

GTR has been frequently used as contemporary dental treatment options in the areas of maxillofacial and oral surgery, implants, and periodontology. 32 In this study, we utilized bibliometric approach to examine the scientific literature published on GTR to identify top authors, nations, journals, and institutions. This will provide researchers more precise study directions, This is because by applying bibliometric analysis, clinicians and researchers can acquire high-impact content through identification of influential author's significant contribution in leading journals.

### Trend of Publication

The first scientific literature on GTR was published in 1987, in the *Journal of Clinical Periodontology* , and had been cited 72 times. 33 This paper, a clinical trial, was conducted based on the principles of GTR to assess the regenerative potential of the periodontal tissues in case of grade II and III furcation defects in mandibular molars. The first author for this scientific literature was R. Pontoriero, who has also been identified as the leading author in this domain. Though with course of time publication increased significantly in number from 1995 till 2004. During 2001, 12 noteworthy papers were published. Among them, the paper by Pintippa Bunyaratavej, in the *Journal of Periodontology* attained 416 citations. This was a highly cited publication. 34

### Leading Countries

The findings from the current analysis revealed that the most of scientific contributions were from the United States, which was consistent with outcomes of the past bibliometric analyses of literature. 18 30 The significant contribution made by American authors are primarily due to greater scientific population, active researchers, and abundant financial resources. 24 Moreover, American researcher named Prichard, pioneer of periodontology in the United States, made significant contribution during the development of GTR technique. He contributed to the development of the “pushback procedure,” which involved deepithelializing gums to hinder their rapid migration and help with the healing of periodontal wounds. 35 Other than America, significant contribution has also been made by various other countries, which included Italy, Germany, and Switzerland; this identified the growing research trend in this domain in the European countries. Italian authors made substantial contributions through the development of protocols for reconstructive surgery, to which several very productive authors contributed. 36

### Journals

Eugene Garfield developed the protocol for detecting the IF of any journal. It has been made available by the “Journal Citation Reports (JCR).” JCR provides an essential guide which measures for impact and functioning of each scientific journal. So, IF has been considered as an important tool, which has not only remained useful in bibliometric analysis but also preferred by the scientific society. 37 It has been observed that mostly authors prefer to submit their scientific publications to journals having high IF. 38 Likewise, findings of this analysis revealed that the highest numbers of papers were published in the leading journals, whose IF demonstrated the effect of these journals, and the potential influence of the articles published in them. 39 Furthermore, the year of publication is an additional influencing factor on the number of citations an article obtains, as older papers generally receive higher citations as compared with recent publications. 40

Scimago Journal & Country Rank (SJR) has categorized all the journals listed in their databases into different quartiles. Q1 represent the topmost 25%, Q2 stands for journals present in the 25 to 50% of the group, while Q3 and Q4 represents 50 to 75% and 75 to 100% of the journals. In the same context, the *Journal of Periodontology* and the *Journal of Clinical Periodontology* published highest number of scientific papers. Both journals were published by Wiley and had Q1 ranking. Additionally, it was observed that 9 out of 12 journals had high IF, this further identified the trend that authors chose to publish their papers in high IF journals. These findings are in line with prior bibliometric analysis, where similar trend had been observed. 27

### Leading Institutes and Authors

The University of Bern from the Netherlands published the highest number of scientific literatures related to GTR, followed by Bologna University from Italy. Most of the institutes were from the United States and European countries. However, the University of Siena in Italy attained the highest citations. This identified the presence of ample funding resources, facilities, and availability of highly trained and competent scientists capable of carrying out research of high quality.

With reference to highly cited authors, both Pontoriero Roberto and Pierpaolo Cortellini published 4 papers which had been cited more than 400 times. Both authors made significant contribution in this field.

### Highly Cited Papers

The first highly cited paper was published by Pintippa Bunyaratavej in 2001 in the *Journal of Periodontology* . This paper addressed the significant role and properties of collagen membrane in various periodontal surgical procedures, namely, GTR and guided bone regeneration. Various benefits of collagen in medicine and dentistry were discussed which comprised of healing, stability, and coverage to wound. Capability of crosslinking in collagen has been noted which reduces degradation process, allowing cells to repopulate the regions. Hence, the study concluded that further research was required to study the role of collagen in complete regeneration. 34

The second highly cited paper was published by author Mario Roccuzzo, in the *Journal of Periodontology* . This study evaluated the efficacy of periodontal plastic surgery as a therapeutic option for providing root coverage in gingival recession. This review compared four surgical procedures, which included GTR, coronal flap, free gingival, and connective tissue graft. With respect to GTR, gingival reduction was minimal, while attachment gain proved similar in all procedures. 41

The third paper which attained high citations was a meta-analysis published in the *Journal of Periodontology* in the year 2001 by Eli E. Machtei. This paper aimed to determine the regenerative potential of performing early membrane exposure in GTR and guided bone regeneration. Hence, for this, this study focused on studies which assessed GTR in intrabony and furcation defects, and guided bone regeneration surrounding the implants. It was noted that membrane exposure during healing did not impact GTR surrounding natural teeth. 42

## Keywords

In scientific literature keywords are contemplated as the basic components which convey notions in bibliometric research and have been widely employed to identify study areas. 43 Scientific trends based on the frequency of keywords used, research theme founded on principle of coword clustering, and knowledge mapping using coword networks are assessed. 44 In the current study, numerous keywords were identified, however, among those, GTR, periodontal disease, and membrane were the most frequently selected keywords which had been used by American and European authors.

## Limitations

This study had few limitations. Only original research articles and review papers indexed in the Scopus database were included. Hence, there is a likelihood that literature published in other databases which comprises of WoS, Google Scholar, and PubMed may have been missed out. Second, all the articles were manually searched on Scopus, and there is a possibility of missing out important literature. English was selected as the medium of language and publications in other languages have been overlooked.

## Conclusion

The present study provided useful data concerning the number of scientific articles published from 1987 to 2023. The current analysis also demonstrated significant contributions made by the United States and the University of Siena from Italy as the most active country and organization, and noted the *Journal of Periodontology* as the leading journal which has contributed tremendously on GTR. Despite the decline in the number of publications in the last few years, it is envisaged that this study will enable upcoming and established academics and researchers to construct future possibilities for scientific cooperation and collaboration for research in this domain.
